# Atropia in Ophthalmic and Aural Surgery

**Published:** 1872-06

**Authors:** 


### ATROPIA IN OPHTHALMIC AND AURAL SURGERY.

Dr. Ernest Hart, Ophthalmic and Aural Surgeon to St. Mary's Hospital, writes in the *British Medical Journal:*

"Looking through a long list of cases, I have employed atropia drops in the course of the treatment of 1,523 out of 2,710 consecutive cases of disease and injury of the eye (a list not including, of course, affections of the ophthalmic appendages); and I pass in review a long array of cases in which I had to regret that it had not been used before the patients came into my hands. Two of the most recent consultations which I have entered were cases of injury to the eye during the last month. In both cases, the treatment had been unexceptionable, except that atropia had not been used; and though the globes have been saved, the fixed and closed irides will make the posssibilities of vision depend upon iridectomy under not very favorable circumstances. In the very last consultation on a case of iritis, the treatment had been equally conducted without dilating the pupil. Mercury, leeches and lotions were in use. A darkened room and an antiphlogistic diet were all in use when I was called; but the vision was extremely misty, the pupil contracted, and the patient in proportionate danger of 'false pupil' from the effusion of lymph and of loss of sight.

"Now, I do not think that I shall be using an exaggerated form of expression, or going beyond the strict and well-balanced weight of words which is necessary to give due force to the fact to be conveyed, if I say that we could, in the treatment of ophthalmic disease, better afford at this day, so far as our knowledge of disease and means of mastering it extend, to dispense with all other drugs, lotions and applications put together, than with this one *topical* medicament. Let us consider what atropine does for the inflamed eye. It allays local sensitiveness and removes local spasm; it gives to the eye and to its internal muscular apparatus—iris and ciliary muscle—physiological rest, the greatest of all curative means. Nor does it do this only; but, in dilating the pupillary aperture, it drives far from us the bugbear which long haunted the ophthalmic surgeon, and which still pursues those who are not sedulously active in the use of atropia—closure of the contracted pupil by an adherent plug of lymph, and gluing of the uveal surface of the iris to the lens. It would rob the consulting-surgeon of a great many profitable but trying operations if the atropine eye-drops were ready in every surgery, not only on all emergencies, but for the exigencies of a daily practice. It is as safe a rule, in ophthalmic practice, to use an atropine drop when in doubt as in whist to play a trump. I can hardly think of more than one absolute contraïndication, and that is the existing oval dilatation of the iris in glaucoma.

"There are, of course, also mechanical contraïndications—as in peripheral wounds of the cornea, with hernia of the iris, where to dilate the iris, is to increase the peripheral protrusion; but even here, the moment the corneal gap is healed, atropia becomes of the first necessity. But in all cases of iritis, in contusions and injuries of the eye, in corneitis, purulent ophthalmia, scrofulous ophthalmia, and deep-seated mixed inflammations of the eye, the local instillation of a solution of atropia is the most precious of therapeutic means. The most useful strength is, I think, expressed in the formula: ℞—Neutral sulphate of atropia gr. ij; glycerine, gtt. v; distilled water ℥j. The frequency of the use of the drop must vary with the facility and rapidity with which it acts. Where the iris has become much inflamed before the local treatment is adopted, it is sometimes very indocile, slow to respond, and hard to dilate. Then the measure of frequency must be the amount of resistance, and perseverance must be the rule of treatment. In the treatment of keratitis and minor cases of deeper inflammations, one application a day, or at most two, will suffice; and presently, once in two or three days. It will be enough, then, to keep the pupil dilated, and the ciliary apparatus at rest and free from tormenting spasm. The present result of the most careful observation of the origin and cause of ophthalmic disease, pursued with the advantages of the improved methods of optical diagnosis now at our command, is to simplify our treatment, and to ostracise a majority of superfluous agents of medication. With a little cotton-wool, alum, and glycerine, hot and cold water and atropine, and a pocket-case of instruments, we can treat, with a previously unattainable success, nearly the whole range of ophthalmic diseases. I am speaking of local and surgical treatment. Ointments, poultices, caustics, irritants, scarifications, venesections, blisterings, and setons, may be looked upon almost as things of the past. The medical, dietetic and hygienic treatments are favorably modified, and simplified to an almost equal extent, by the intelligent study of diathetic indications; but the whole shopful of topical applications may be left aside by the surgeon who will thoroughly apply the vast resources of the few simple agents I have named; and of them all, atropine is greatest.

"I will not undertake to say that mercury is useless in the treatment of some forms of (syphilitic) iritis; but I will affirm that I have repeatedly seen iritis occur and run a very severe course in patients previously and at the time already under the influence of mercury, and that in a long series of cases which I treated by atropia and careful dieting only, and without mercury, during five years at St. Mary's Hospital, the results were so excellent that I could not affirm that they would have been improved by the most guarded and judicious use of mercury.

"It is possible, though not easy, to abuse atropine. It must not be used, as I have said, in glaucoma or in peripheral wounds of the cornea. A case or two of troublesome constitutional symptoms, through absorption of the excess by the lachrymal mucous membrane, have been recorded. This is never likely to occur with ordinary care, and I have never seen it occur, but it may be well to bear it in mind in treating infants. The most convenient and unfailing application may be made by the use of the atropized gelatine disks which I introduced a few years since, and which are now largely used in this country and abroad. They are always ready, do not spoil by time, and are precisely dosed, each disk containing as much as a drop of the solution I have mentioned."—*American Practitioner.*

